# Supplementary material for: Cheating leads to the evolution of multipartite viruses
Source: PLoS Biol. 2023 Apr 24;21(4):e3002092. doi: 10.1371/journal.pbio.3002092 (PMC10159356; doi:10.1371/journal.pbio.3002092)
Supplement: S1 Text — Supporting information file contains Figs A–E, and Tables A and B, together with a legend for each supplementary Figure and Table. (DOCX) [file pbio.3002092.s001.docx]

# Supporting Information for ‘Cheating Leads to the Evolution of Multipartite Viruses’

| Figure A: Analytical Model Dynamics |
| --- |
|  |
| 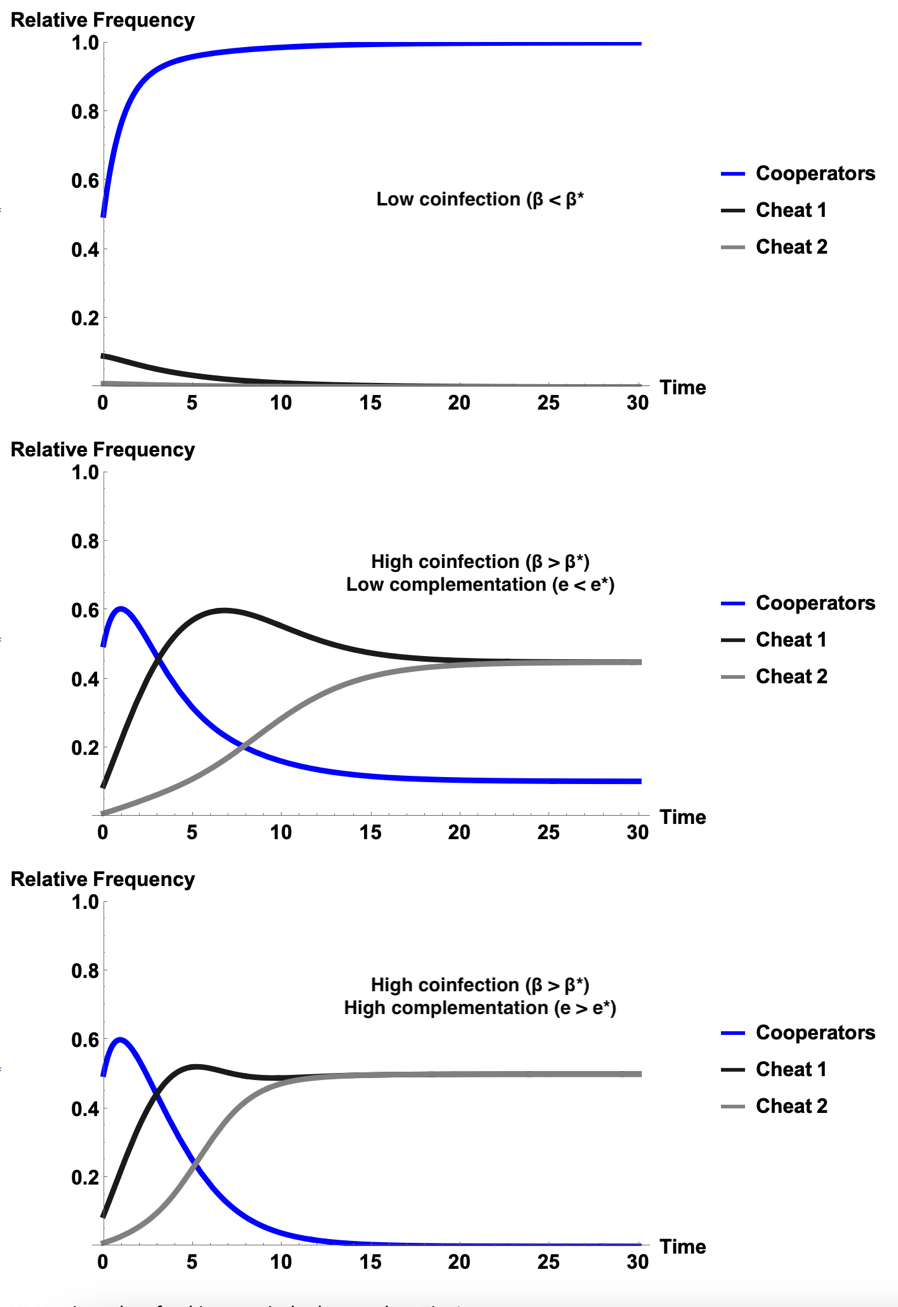 |
|  |
| Figure A: Negative frequency dependence in the analytical model. We plot the dynamics of the three potential strategies when the model is solved numerically using replicator dynamics for different parameter combinations, to illustrate three key types of outcome (Equations 1.1-1.3). (a) When the rate of coinfection ($\beta$) is too low, cheats cannot invade (parameters: a=0.5, b=2, c=0.1, d=1, e=0.25, $\beta$=0.25). (b) When the rate of coinfection ($\beta$) is high enough, cheats can invade. If the possibility for complementation between cheats (e) is below a threshold e*, cheats then coexist with cooperators (parameters: a=0.5, b=2, c=0.1, d=1, e=0.25, $\beta$=0.8). (c) If the rate of coinfection ($\beta$) is high enough, and the possibility for complementation between cheats (e) is high enough, cheats invade and then replace cooperators (parameters: a=0.5, b=2, c=0.1, d=1, e=0.75, $\beta$=0.8). This Figure can be generated using the data and code at <https://doi.org/10.17605/OSF.IO/PBE4N> |

| Figure B: Negative frequency dependence |
| --- |
| 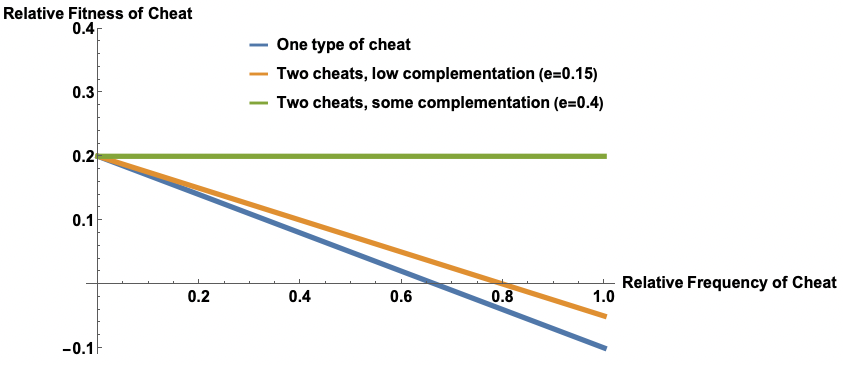 |
|  |
| Figure B: Negative frequency dependence in the analytical model. When the possibility for complementation between cheats is low or non-existence (low e), the fitness of cheats decreases as cooperators become rarer, and so cheats and cooperators coexist. However, as the possibility for complementation (e) increases, the fitness of cheats becomes less dependent on the abundance of cooperators. When complementation is high enough, cheats are able to drive cooperators extinct. This Figure can be generated using the data and code at <https://doi.org/10.17605/OSF.IO/PBE4N> |

| Figure C: Multipartitism evolved under a range of parameters in the simulation |
| --- |
|  |
| 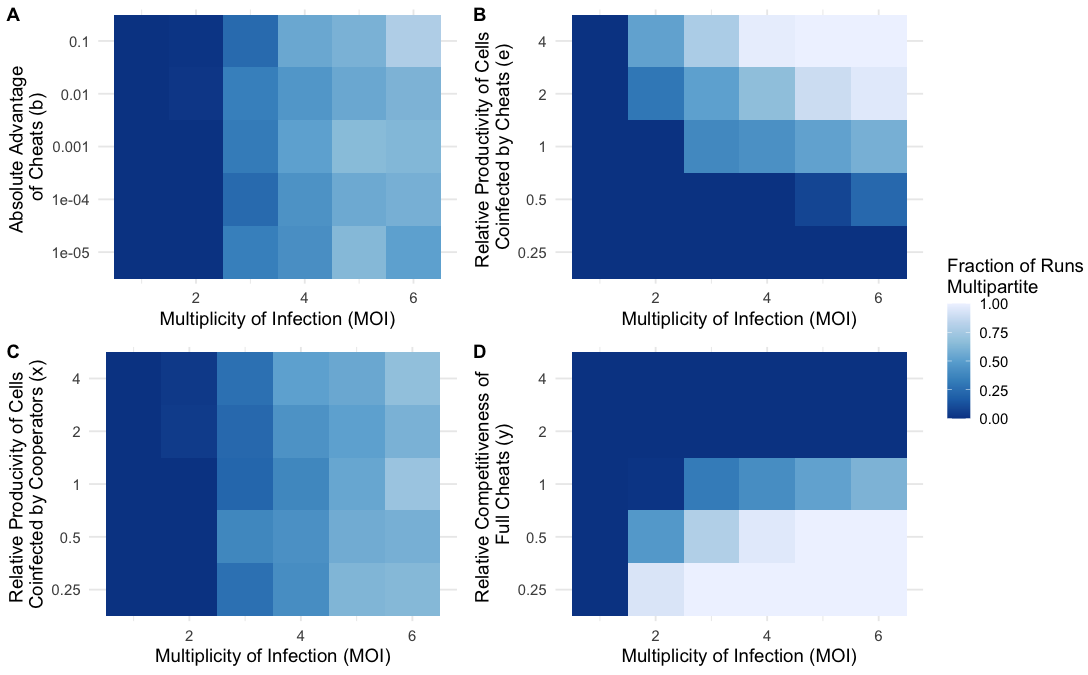 |
| Figure C: Multipartitism evolves over a range of conditions in the simulation. Each block in the heatmap represents the fraction of simulation runs resulting in a multipartite viral population; lighter blues indicate that a higher fraction of runs resulted in multipartitism. This Figure can be generated using the data and code at <https://doi.org/10.17605/OSF.IO/PBE4N> |

| Figure D: Group Benefits and the Evolution of Multipartitism |
| --- |
|  |
| 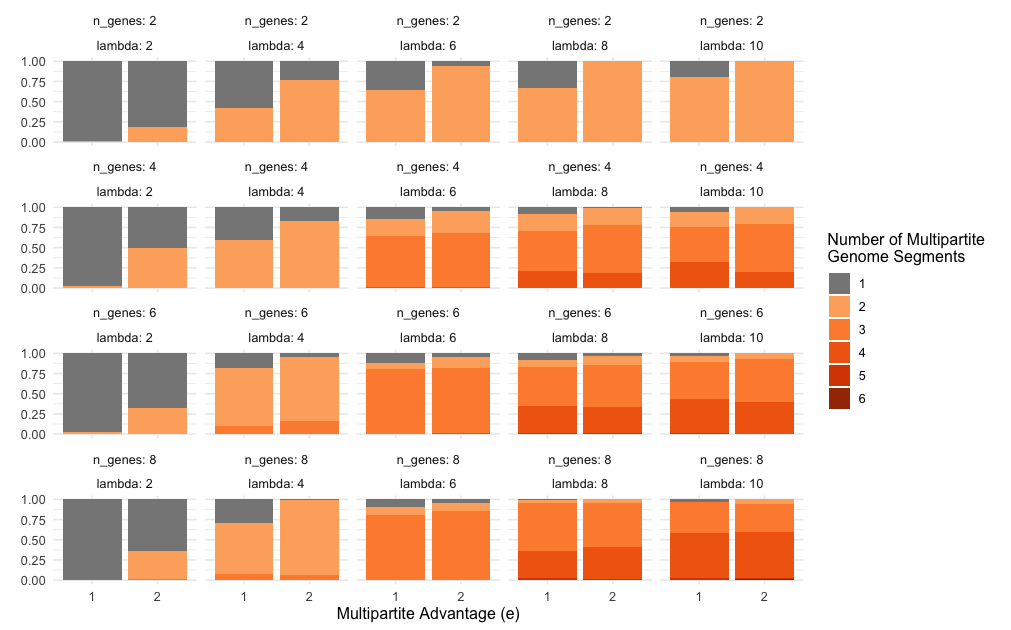 |
| Figure D: We found that including a relatively large advantage to multipartitism (*e=2*) only marginally increased the extent to which multipartitism evolved, and only for genomes with a small number of genes, primarily when coinfection was rarer (lower lambda). This Figure can be generated using the data and code at <https://doi.org/10.17605/OSF.IO/PBE4N> |

| Figure E: Multipartitism evolves more easily with a larger genome |
| --- |
| 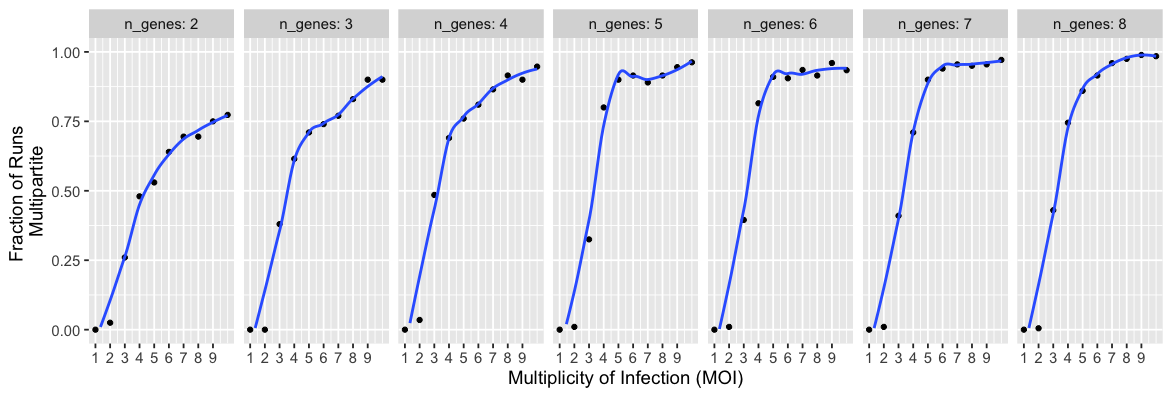 |
|  |
| Figure E: Multipartitism evolves more easily with a larger genome. When there were more genes in the viral genome (different panels), a larger fraction of simulation runs resulted in a multipartite viral population. Here, we excluded simulation runs that resulted in a population crash, and each point reflects 500 simulation runs. This Figure can be generated using the data and code at <https://doi.org/10.17605/OSF.IO/PBE4N> |

Table A: A list of the viral genera recorded to be multipartite, with the number of multipartite segments, associated taxonomic data, a record on whether defective interfering genomes have also been recorded in each genus, and the number of studies we found mentioning that viral genus. References and analysis details are available in Appendix 4 and the associated supplementary material. This Figure can be generated using the data and code at <https://doi.org/10.17605/OSF.IO/PBE4N>

| \| Virus \| Size of cheat deletion \| Cooperator fitness alone (a) \| Cooperator fitness vs cheat (c) \| Fraction of cells coinfected for multipartitism, assuming no benefit to multipartitism (e=1) \| \| --- \| --- \| --- \| --- \| --- \| \| poliovirus \| Large \| 0.5025 (1) \| 0.0040 (2) \| 0.5032 \| \| VSV \| Large \| 0.6037 (3) \| 0.0857 (4) \| 0.5930 \| \| VSV \| Large \| 0.6037 (3) \| 0.0100 (3) \| 0.5520 \| \| VSV \| Large \| 0.6037 (3) \| 0.0001 (3) \| 0.5470 \| \| Rabies (small plaque variant) \| Large \| 0.6037 (3) \| 0.0200 (5) \| 0.5571 \| \| Rabies (large plaque variant) \| Large \| 0.6037 (3) \| 0.0110 (5) \| 0.5525 \| \| Bunyamweravirus \| Large \| 0.5490 (6) \| 0.0970 (7) \| 0.5767 \| \| Phage Phi6 \| Small \| 1.0000 \| 0.6500 (8) \| > 1 \| \| phage ms2 \| Small \| 1.0000 \| 0.9000 (9) \| > 1 \| | |
| --- | --- | --- | --- | --- | --- | --- | --- | --- | --- | --- | --- | --- | --- | --- | --- | --- | --- | --- | --- | --- | --- | --- | --- | --- | --- | --- | --- | --- | --- | --- | --- | --- | --- | --- | --- | --- | --- | --- | --- | --- | --- | --- | --- | --- | --- | --- | --- | --- | --- | --- | --- |
| Table B: Cheats can drive the evolution of multipartitism under realistic conditions. We used existing experimental data to derive estimates for the parameters in our analytical model (Methods). We then used these parameters to determine whether our model would predict the evolution of multipartitism for cheats that had similar properties to those described experimentally. A detailed table with calculations for each estimate is available with the supplementary material. This Figure can be generated using the data and code at <https://doi.org/10.17605/OSF.IO/PBE4N> |  |

# References

1. Flint J, Racaniello VR, Rall GF, Skalka AM. Principles of Virology [Internet]. 4th ed. American Society of Microbiology; 2015 [cited 2018 Sep 27]. Available from: http://www.asmscience.org/content/book/10.1128/9781555819521

2. Shirogane Y, Rousseau E, Voznica J, Xiao Y, Su W, Catching A, et al. Experimental and mathematical insights on the interactions between poliovirus and a defective interfering genome. PLOS Pathog. 2021 Sep 27;17(9):e1009277.

3. Akpinar F, Yin J. Characterization of Vesicular stomatitis virus populations by tunable resistive pulse sensing. J Virol Methods. 2015 Jun 15;218:71–6.

4. Huang AS, Wagner RR. Defective T particles of vesicular stomatitis virus: II. Biologic role in homologous interference. Virology. 1966 Oct 1;30(2):173–81.

5. Kawai A, Matsumoto S. Interfering and noninterfering defective particles generated by a rabies small plaque variant virus. Virology. 1977 Jan 1;76(1):60–71.

6. Lowen AC, Boyd A, Fazakerley JK, Elliott RM. Attenuation of Bunyavirus Replication by Rearrangement of Viral Coding and Noncoding Sequences. J Virol. 2005 Jun;79(11):6940–6.

7. Patel AH, Elliott RM. Characterization of Bunyamwera virus defective interfering particles. J Gen Virol. 1992 Feb 1;73(2):389–96.

8. Turner PE, Chao L. Prisoner’s dilemma in an RNA virus. Nature. 1999 Apr 1;398(6726):441–3.

9. Meir M, Harel N, Miller D, Gelbart M, Eldar A, Gophna U, et al. Competition between social cheater viruses is driven by mechanistically different cheating strategies. Sci Adv. 2020 Aug 1;6(34):eabb7990.
