# Supplementary material for: Cheating leads to the evolution of multipartite viruses
Source: PLoS Biol. 2023 Apr 24;21(4):e3002092. doi: 10.1371/journal.pbio.3002092 (PMC10159356; doi:10.1371/journal.pbio.3002092)
Supplement: Attachment — Submitted filename: genome_splitting_reviews_response_23_02_03_submission_pdf.pdf [file pbio.3002092.s002.pdf]

As you will see below, the reviewers are mostly positive about your study, but each raises a few concerns that must be addressed before further consideration. As the Academic Editor summarised, "The paper is well written and interesting, yet I agree with the reviewers that especially the relation to the data should be made more explicit to warrant publication in PlosBio. I also think that Reviewer 1 is right that some generalizations of the model may not be too difficult. While I agree with Reviewer 2 that the manuscript should not be published in PlosBio in its *current* state, I am optimistic that the authors can improve it much by addressing the reviewer comments."

Dear Editor,

Thank you for your careful consideration of our article. We have thoroughly revised the article in response to the reviewers' concerns. In particular, we have extended the model in two ways: firstly to include the possibility for multiple genomes to be packaged inside the same virion (as suggested by all three reviewers); and secondly to include a detailed analysis of a novel adaptive benefit of multipartitism, in reducing exploitation by full cheats (as suggested by Reviewer 2). As a result of this, we have restructured the manuscript to include two new figures, reworked versions of most existing figures, we have incorporated greater links to data throughout, and we have addressed the framing concerns raised by the reviewers.

Please find below our detailed response to reviewers. Text changes to the main manuscript have been highlighted in red.

Reviewer #1:
[identifies himself as Eugene V Koonin]

The paper by Leeks et al explores a long-standing evolutionary problem, the origin of multipartite viruses (with implications for other genome segmentation phenomena). The authors develop a game theoretical model under which multipartitism can evolve through cheating alone, without any group benefits.

This approach is commendable because as a general principle, the possibility of non-adaptive origin of any phenomenon should be explored before adaptation is invoked. On the whole, the results of the model analysis are convincing. This is valuable and interesting work.

We thank Professor Koonin for his careful reading of our manuscript and kind words.

However, I believe the model fails to incorporate a fairly obvious and general advantage of genome segmentation. Different segments of multipartite genomes can be translated, transcribed and replicated simultaneously, resulting in increased rates of each of these processes, under the realistic assumption that the amounts of the enzymatic machineries involved are sufficient. I think a parameter capturing this enhancement of genome expression and replication should be included in any model. This will not invalidate the conclusion that cheating is at the root of multipartitism but is likely to help explain why genome segmentation is often fixed in evolution despite the costs. Many monopartite riboviruses of eukaryotes have evolved mechanisms of subgenomic RNA synthesis, circumventing the problem of expressing multiple genes from the same genome (mostly pertinent for viruses of eukaryotes in which indeed multipatitism is common). This is an evolutionary strategy

that represent an alternative to multipartitism and probably should be discussed as such. Further, both multiparititims and sgRNA formation (and also programmed frameshifts in some riboviruses) provide the important opportunity for differentially regulating the production of virus proteins. It is certainly not chance that in the great majority of multipartite viruses, genes coding for replication system components and structural proteins are partitioned between different segments. All in all, I agree that cheating is at the root of multipartittims, but I believe that its widespread fixation in virus evolution also has adaptive components to it, I think this should be taken into account both in the model itself and its interpretation and discussion.

We thank Professor Koonin for this interesting hypothesis concerning simultaneous transcription and translation. We agree that this seems like a highly plausible mechanism by which segmented and multipartite viruses could gain an advantage, and that this kind of mechanism may especially play a role in the evolutionary maintenance of multipartite viral genomes.

In our model, higher values of our parameter 'e' allow cells infected by multipartite viral genomes to produce more virions than cells infected by monopartite viral genomes. Hence, this parameter captures the net effect of different types of mechanistic advantage that multipartitism could involve, such as the potential for simultaneous transcription and translation, while leaving out an explicit consideration of the intracellular dynamics.

In response to this comment and others, we have added:

- A new subsection on group benefits and the evolutionary maintenance of multipartitism in the discussion (lines 594-629). In this, we explicitly consider the extent to which our 'e' parameter captures different mechanistic advantages to multipartitism. In particular, in lines 602-611 we point out the limitations of our 'e' parameter in terms of capturing the advantage of simultaneous transcription/translation.
- A new supplementary figure (Fig. S4) in which we explore the relationship between cheating and group benefits in driving the evolution of multipartitism. We have added a new paragraph to the main text to discuss this relationship (lines 347-355).
- A new figure (Fig. 5) and a new results section (lines 401-435) in which we discuss a new type of benefit to multipartitism that emerges from our model, that could be adaptive at the group level.

As we mentioned in our response to Reviewer 2, a key goal of our paper is to stimulate further research, especially concerning the mechanisms that might allow for the evolutionary maintenance of multipartitism. We hope that the additions we have made in response to this comment will be fruitful to this end.

Another point pertains to viruses with multiple genome segments packaged within the same particle. In the current manuscript, these are just briefly mentioned towards the end of the Discussion. However, it seems to me that these can be smoothly included in the same modeling framework by replacing the cost and rate of coinfection with the corresponding values for genome segments assembly in virions. The choice of one strategy over the other during evolution will depend on the rate of coinfection, and this can be both modeled and gleaned from the available empirical data.

We have now extended our model to include the possibility that multiple viral segments are encapsidated inside the same virion (Fig. 6). As Professor Koonin predicted, this has allowed us to show that our mechanism could also explain the evolution of segmented viruses.

There are a number of assumptions involved in building this extension into the model. These assumptions suggest some useful further extensions of our model. For example, virion packaging can be selective or non-selective, can involve different numbers of viral genomes, can involve producing virions with a greater total amount of genetic material (hence requiring physically larger virions), can be a fixed feature of the virus or of the host cell or can be an evolvable feature, or can co-evolve alongside other viral strategies such as cheating. It was beyond the scope of this paper to explore all the possible ways that genome segmentation could occur. Therefore, we have also added a new section in which we discuss the evolution of genome segmentation (lines 631-758). In this, we discuss our results on the evolution of genome segmentation in relation to what is known about the relevant biology of different systems (lines 639-686), and we explore how the assumptions we have made could be changed to reflect the biology of different viral systems (lines 691-698).

We think that these substantial additions strengthen the manuscript, and so we thank Professor Koonin for this useful suggestion.

The rest of my comments pertain to presentation.

The current Introduction is rather verbose, but also somewhat too general. I believe the paper would benefit a lot from a more specific coverage of the actual data on virus multipartitism. Ideally, even a supplementary table containing such data across the virus realms, kingdom and phyla would strongly enhance the paper and help build the case for the major biological impact of multipartitism.

This is a good point. We have now:

- Included extra detail on the prevalence and abundance of multipartitism in the Introduction (lines 36-49).
- Produced a supplementary table with data on multipartitism across different viral taxonomic groups, and referenced this in the Introduction (Table S1).
- Added additional references to several recent comprehensive reviews on this topic, which explore the taxonomic breadth of multipartitism in more detail than we are able to in our review (lines 36-49; 631-686; and elsewhere).

In the Discussion, I believe it would be helpful to refer the Constructive Neutral Evolution concept that I believe is directly relevant for the non-adaptive origin of multipartititsm.

We now cite “Stoltzfus, A. 1999. On the Possibility of Constructive Neutral Evolution. J Mol Evol 49: 169–181.” at the relevant point in our Discussion (line 629).

The authors refer to the "lack of well resolved tree of viruses", citing Walker et al 2020. This is quite misleading as written. On the one hand, there is no such thing as an evolutionary tree of all viruses, but on the other hand, for many groups of viruses, the trees are quite well resolved This has to be rephrased.

We have fixed this (lines 475-476).

The authors are unduly fond of the phrase "in principle" - I really think it should be minimized if not

completely eliminated.

We have removed all three instances of “in principle” from the manuscript.

Reviewer #2:

In this article, the authors aim to better understand the existence conditions of multipartite viruses (hereafter:, mp viruses). They discuss existing hypotheses in the field, namely i) group benefits, ii) intragenomic conflicts between segments, and iii) small particles being favoured (e.g. bij persisting longer). The authors discuss how these hypotheses are limiting, as many mp viruses have 3 or more segments, so the opportunity costs are subsential while the imagined benefits are marginal. They support their skepticism well by stating that existing models requires incredible high rates of coinfection (up to 100) while in reality no more than 2-13 have been observed. The authors therefore challenge existing models by envisioning a hypothesis of their own: gene products of viruses are public goods, opening the door to cheats. Work has indeed shown that cheats can grow much faster than full genomes. As I understand it, mp viruses are, in the author's view, the result of "an evolutionary race to the bottom", or "tragedy of the commons".

The manuscript is very well written, and I found only one minor error (see below).

We thank Reviewer 2 for their kind comments.

However, while I am happy to consider the hypothesis by the authors, I find their methods and the analysis of the models lacking.

We thank Reviewer 2 for their careful reading of our manuscript. In our view, Reviewer 2’s major comments primarily concern interpretation and presentation, rather than methods and analysis. We have now made the suggested changes, and we think that these have improved the presentation of the manuscript.

My biggest concern is that that authors phrase an inevitable consequence of cooperator-defector dynamics to be the one and only explanation needed to understand mp viruses. At numerous (at least 5) points in the text, they found it necessary to discuss how their model is superior because it does not require group benefits. In one occasion in particular, it was even phrased in such a way as to suggest no further work was needed: "Consequently, our work does not require new kinds of mechanism to be uncovered in order to explain multipartitism, nor does it require searching for elusive group benefits to multipartitism." In my opinion, this is in very poor form, where it could even suggest that "we know it all now". This is not productive, and not helpful to scientific discourse.

We did not intend to present our model as the one and only explanation needed to understand multipartite viruses. Our argument is that cheating relies on existing known mechanisms and has been widely demonstrated in a range of viruses. Hence, cheating is more parsimonious than explanations that require new mechanisms that have not yet been discovered.

We thank Reviewer 2 for pointing out how our language could be misinterpreted here. We have made many changes in response to this comment:

- We have rewritten much of the Discussion in order to be more explicit about the ways in which our model differs from existing models, while being careful to make sure our work can't be misinterpreted as implying that it is 'superior' (lines 498-501; 563-564; 568; 594-596; 632-637; and elsewhere).
- We have added a new supplementary figure (Fig. S4) in which we explore the relationship between cheating and group benefits in driving the evolution of multipartitism. We have added a new paragraph to the main text to discuss this relationship (lines 347-355).
- We have added a new section in the Discussion on the potential for group benefits to explain the maintenance of multipartitism (lines 594-629). In this section, we discuss the potential for our model to incorporate different types of group benefit.
- We point out numerous explicit changes and extensions to our model that would be useful. These include changes to incorporate additional group benefits (lines 602-611) and changes to incorporate different assumptions about genome packaging (lines 689-698).
- Inspired by Reviewer 2's comment above, and in order to make sure that our work is not misinterpreted as suggesting that 'we know it all now', we explicitly point out a number of future directions that should be investigated, with respect to the role that group benefits may play in the evolutionary maintenance of multipartitism (lines 602-611 and 624-629).

The authors move on to make multiple predictions based on their game theoretical model, where mp evolves if:

1. coinfection is common
2. advantage of cheating is high
3. high e (which honestly, is an assumption that requires unpacking!)

We have added a section that focuses on group benefits to multipartitism in the Discussion. In this, we unpack the kinds of advantage that are captured by a high 'e' or not (lines 594-629).

4. cooperators are strongly outcompeted within their cells (which may be a strong assumption, but I grant you this given the 10,000 fold benefit discussed in the intro)

This is an assumption that varies between different types of viral cheat. In Table S2 we present seven examples of viruses in which cooperators are outcompeted more than 100-fold, and two examples in which cooperators are outcompeted less than this.

In Figure 3, we now include viruses where cheats gain a large advantage, and where they gain a smaller advantage. We have also added additional citations to our Table S2 throughout this section of the text.

5. cooperators do worse alone than with another cooperator (is this a fair assumption?)

This is a common finding in viruses and elsewhere, hence we think it is a useful model result to report. Some specific evidence for $d > a$ in viruses comes from:

- Stiefel 2012 (http://dx.doi.org/10.1021/nl3018109) finds that a cell infected by two vaccinia viral genomes is more than three times as likely to be successfully infected, compared to a cell infected by one vaccinia viral genome;
- Andreu-Moreno et al 2020 (10.1126/sciadv.abd4942) suggests that the total viral genome production per capita is more than ten-fold higher for cells infected by two viral genomes,

compared to cells infected by one viral genome, across VSV, Influenza A, human Adenovirus A, RSV, CVB3, and Vaccinia;
- Andreu-Moreno and Sanjuán 2018 find that the total viral genome production per capita is roughly three times higher for cells infected by two VSV genomes compared to just one VSV genome (10.1016/j.cub.2018.08.028).

We have now changed this text to be clearer about this model prediction (lines 224-225). We also now discuss the relationship between a and d more explicitly when introducing d in the main text, including referencing the above three empirical studies (lines 180-183). Finally, we have changed the text in this section to be clear that these factors make multipartitism more likely to evolve, but none of them are strictly necessary for its evolution (line 217).

Generally, I found the predictions marginally insightful, as most of them came down to a cost-benefit analysis without any mechanistic insight as to what drives the costs- and benefit-parameters in the first place.

We agree that game theory models depend upon cost/benefit analyses. However, while this is limiting in some ways, it is also a strength, as it allows predictions to be made that are not dependent on specific mechanistic details, that might only apply in some biological systems. Therefore, we think this is a useful way to model a phenomenon such as multipartitism, which occurs across varied biological systems. Similar game theory models have been used broadly to model viral cheating in the past, and we chose to use a game theory approach partly in order to be consistent with those models (e.g. Turner & Chao 1999, 2003; Chao and Elena 2017; Meir et al 2020; Nee 1987; Chao 1991).

Nevertheless, at various points in the manuscript, we do go into mechanistic details of how these benefits arise in viruses, as far as the existing data allow us to:
- We have changed Fig. 3 to include two examples of ‘small deletion’ cheats, that do not gain sufficient advantages to lead to multipartitism via our model
- We have added new sections to both the results (lines 401-435) and the discussion (lines 594-629) on group benefits to multipartitism. In the discussion, we provide mechanistic insights into the elements of group benefit that our model does or does not capture (lines 604-611).
- In lines 545-560, we provide explicit mechanistic discussion of how viral cheats can gain advantages though: shorter length, especially for viruses that use Geometric mode of replication or other non-linear feedbacks; out-competing cooperators for entry into virions; out-competing cooperators for access to replicase enzymes; and modifying their genomes in ways that were not possible for cooperative viruses. Further mechanistic details on the advantages that viral cheats gain can be found in the recent reviews on this topic that we reference (e.g. Leeks et al 2021; Vignuzzi and López 2019).

We would be keen to include further mechanistic details of cheat/cooperator interactions in viruses, if the reviewer feels we have left out specific concepts.

For example: "Our model predicts when cheats are highly competitive relative to cooperators [...], cheats evolve. This is not particularly insightful.

We did not say this. The full excerpt from the text was: “For example, our model found when cheats are highly competitive relative to cooperators within coinfected cells ($c \sim= 0$), and

when cells infected by one cooperator are half as productive as cells infected by two cooperators ($a=d/2$), multipartitism evolves when half of cells are coinfected ($\beta = 0.5$), even in the absence of any group benefits to being multipartite ($e=d$)."

The key insight from our model is not to uncover when cheats evolve (this was already known), but to uncover when multipartitism evolves via cheating (this was not known). In the sentence being quoted, we list some of the conditions in which our model predicts multipartitism evolves via cheating. One of these conditions is that cheats are highly competitive relative to cooperators within coinfected cells. We then dedicate the next section of the main text to unpacking this assumption and investigating when it holds empirically.

To avoid future confusion, we have now rewritten the quoted section of the manuscript (lines 217-242).

The authors attempt to support these insights with figure 3, but to make this analysis somewhat convincing, it should also contain examples of clearly non-mp viruses. The way the data is presented here, any virus could be either mp or non-mp, as the parameter range shown is sufficiently large as to capture all possible outcomes.

We have changed this figure to include two examples of known viral cheats that do not exploit cooperators sufficiently for multipartitism to evolve (Fig. 3). These examples were previously discussed in the main text and Appendix. We thank Reviewer 2 for suggesting that we bring more of our analysis into the main figure in order to make it more convincing.

Perhaps the most insightful one was a brief sentence all the way at the end of the discussion, with a reference to figure S7. The figure itself however is not very well thought through (having a boolean variable on a continuous colour gradient), but I found this figure very interesting, if only it gained half the attention of the rest of the manuscript. I think your work could be much more interesting if you'd consider going into more detail with S7, and perhaps also investigate why many viruses are NOT mp, considering how your analysis suggests this to be a very prominent attractor of virus evolution. I would the analysis w.r.t. the "cheat load" is one that would truly bring your work to the next level (so, S7, but also what is discussed at L397 onwards)

We thank Reviewer 2 for their helpful comments concerning this result. In response to these comments, we have extended the model to include an extensive new analysis of the group benefit to multipartitism via reducing 'cheat load'. This has resulted in a new figure (Fig. 5), and new sections of the Results (lines 401-435) and Discussion (lines 594-629). The additional analysis has revealed a number of insights that we feel may be of interest to this Reviewer in particular:

- The evolution of multipartitism results in a lower abundance of full cheats.
- Splitting into greater numbers of multipartite genome segments results in correspondingly lower cheat load.
- When multipartitism evolves, the details of how many genes are encoded by each segment matters for the corresponding cheat load. When the split more uneven (e.g. one genome encoding 7 genes and one encoding 1), then the cheat load is reduced (Fig. 5). This result occurs because more uneven splits result in higher abundance of the smaller genome segment, meaning that each cell is a more competitive environment, which is then less exploitable by full cheats.
- We have removed the old Fig. S7 and replaced it with Fig. 5.

We feel that these results add a number of new insights resulting from our model, resulting in a new level of analysis regarding group benefits.

Regarding the question of why more viruses are not multipartite, we now consider this question more explicitly in lines 631-758 of the discussion. We especially focus on how our model parameters map onto known empirical trends, such as the relatively high prevalence of multipartitism in plant viruses, and how these factors might be relevant in systems where multipartitism is rarer or less explored, such as temperate phages.

While I actually support the angle that the author take, and found the writing incredibly well thought out, I would not recomment this work to be published in its current state. I want to clarify that I find it really difficult to write such a negative review, and that this is simply my best attempt at trying to help to improve the manuscript for future submissions. I wish the authors the best of luck!

We thank Reviewer 2 for their kind comments regarding the writing of the manuscript, and for their suggestions for improvements that we have now incorporated.

Minor points:

1. Line 96 repeats line 85, quite quickly after the first claim of fitness benefits of cheaters. I don't mind some repetition, but this was so shortly after eachother that I think it can be merged.

We have changed the language here to avoid repetition.

2. Figure 1 has very detailed genomes inside the hexagons while they are really small. Perhaps consider moving the legend some place else so the hexagons can be a bit bigger. Or alternatively, have less detailed genome illustrations to make the figure more readable.

We have enlarged this figure and moved the legend to the bottom to make room (Fig. 1).

3. Payout d is not discussed in the main text, although I could infer it from the figures. Now, the main text discusses payout a, b, c, and e (and not d), which should be resolved.

We now discuss payout d in the main text (lines 180-183).

4. The way the simulations work is not discussed well enough in the main text (or methods). Please provide a summary of appendix 3 in the main text.

We have added a summary of Appendix 3 in the main text (lines 307-314). We have also revised all of our commented R code, MATLAB code, Mathematica code, and Bash scripts, to make sure they are intelligible without specific knowledge of each language.

5. "No other factors need to be invoked." similar to my major concern: this suggests the work is "complete", which it of course isn't. There is plenty to still unpack here.

We have removed this sentence. In our section on group benefits, we emphasise that there is much more to unpack, especially concerning group benefits and the maintenance of multipartitism (lines

594-629). In particular, we now point out explicit future directions at multiple points in the discussion (lines 605-611; 624-629; 693-698), to ensure that our work cannot be misinterpreted as suggesting this question is 'complete'.

6. S7 is called S6 in the caption.

We have removed Fig. S7 and replaced it with Fig. 5.

Reviewer #3:

This manuscript addresses the important and unresolved question of the evolution of multipartite viruses. That cheaters can be at the origin of the evolution of such viral systems is not new, but the key results provided here are indeed novel and original. Some of the conclusions appear highly interesting, contrasting with previous models and current state of the art: i) even highly multipartite viruses can evolve when multiplicity of infection is reasonably low, and ii) with no requirement for group-level benefit.

We thank Reviewer 3 for their kind words.

I have two related major general comments and a list of specific comments that should be addressed to improve the quality of the paper, including for readers that are not deep in theoretical approaches (just like me).

Major comments:

1-In the discussion, the authors indicate that the selective advantage of cheats explains genome fragmentation and that the work presented in the manuscript is thus relevant not only for the evolution of multipartite viruses but also for segmented viruses encapsidating their genome segments together in a single viral particle. While true, this is a serious concern because most earlier attempts at explaining the evolution of multipartite viruses were in fact addressing the genome segmentation but not specifically the separate encapsidation of each segment, which is the essence of multipartitism. In this work, one may wonder whether what is addressed is indeed the individual encapsidation of the segments, as claimed in the title and all along the text through the term "multipartite", or less specifically the genome segmentation. The modelling presented here is in a world where one nucleic acid molecule (whether full length or cheat) is individually encapsidated. What would happen in a slightly more realistic world where encapsidation of more than one viral nucleic acid molecule would be possible, e.g., more than one cheat. With such an additional possibility, could multipartitism evolve, or would the so-called segmented virus packaging several segments together systematically take over?

We agree with Reviewer 3 that our original model focussed specifically on the problem of multipartite viruses and did not explicitly consider segmented viruses. We made this choice because multipartitism is the harder evolutionary problem to explain – the primary cost of multipartitism is usually considered to be the fact that some cells may only be infected by a subset of genome fragments; this cost is not as relevant for segmented viruses. Therefore, we originally wrote our paper with the implicit assumption that if we could explain the origins of multipartitism via cheating,

then cheating could also explain segmented viruses, since these are similar to multipartite viruses but without the same substantial costs.

However, in response to this comment, we have now extended our model to include the possibility that multiple genome fragments are packaged inside the same virion, resulting in a new section of the results (lines 441-466), with a new figure (Fig. 6). We found that genome fragmentation evolves more easily when we allow multiple genome fragments inside the same virion. This occurs because multiple packaging of virions increases the effective MOI of each host cell at the level of the genome (even if the MOI is the same at the level of the virion).

There are many mechanistic assumptions that could potentially go into this extension. For example, virion packaging can be selective or non-selective, can involve different numbers of viral genomes, can involve producing virions with a greater total amount of genetic material (hence requiring physically larger virions), can be a fixed feature of the virus or of the host cell or can be an evolvable feature, or can co-evolve alongside other viral strategies such as cheating. We think that incorporating these details could be a useful extension of our model. Therefore, we explicitly discuss these possible extensions in a new section of the Discussion (lines 688-698).

2-Perhaps related to the above comment, the attempt at extending the results/conclusions presented here to very different systems such as integrated phages, plasmids, and bacterial endosymbionts, is troublesome. It is confusing because these different biological systems are presented as if they all face the same cost ("despite these costs multipartite genomes are found widely throughout nature"). They do not. What they have in common is the fragmentation of the genome but not the separate encapsidation and so not the separate spread of the distinct segments. Endosymbionts of cicadas have split lineages with distinct genome parts, but they are in the same insect cells or bacteriocytes. Each lineage exchange mandatorily with the host but not directly with another lineage, or at least this would need demonstration. Plasmids recombine their replication origins and or can occasionally highjack the conjugation system encoded by another plasmid, but they do not necessarily reciprocally provide complementary functions, so direct comparison with multipartite viruses is arguable, in the least. Integrated bacteriophages are scattered in the same host genome and vertically transmitted together.

We agree that many of these other biological systems may face smaller costs than multipartite viruses, and also that the biological details will matter. For example, as Reviewer 3 points out, integrated bacteriophages will likely be transmitted together when transmitting vertically (analogous to segmented viruses), although they will also transmit separately when transmitting horizontally (analogous to multipartite viruses). We are not claiming that all plasmids and all integrated bacteriophages are analogous, but only the ones which have complementary functions, as given in the references. For instance, in the cicada endosymbiont example, genome splitting has occurred in the sense that a single ancestral symbiont genome has fragmented into multiple genomes, some of which localise in separate host cells (https://www.pnas.org/doi/full/10.1073/pnas.1421386112).

To clarify this, we have rewritten our Discussion to contain a new section, in which we explicitly discuss these biological details (lines 631-758).

Additionally, while our previous modelling only considered multipartite viruses, our model extension, made in response to the previous comment, now shows that cheating can also drive the evolution of genome fragmentation when all genome fragments are cotransmitted (Fig. 6). We thank

Reviewer 3 for the suggestion to extend the model in this way, as we think that this strengthens our Discussion section on genome fragmentation across nature.

The problem is that multipartitism is not clearly defined from the start (first lines of the introduction) as several genome segments (interdependent) encapsidated and propagated/spread separately from cell-to-cell and host-to-host; Or do the authors have another definition? I am not sure that the incurred cost is the same for multipartite viruses, segmented viruses, integrated phages, plasmids, and bacterial endosymbionts, I even clearly doubt it.

We now define multipartitism in the second sentence of the Introduction (line 36-38), as well as in the first line of the Abstract. We agree that segmented and multipartite viruses will face different types of cost, and we now explore this explicitly in the Discussion (lines 688-698).

These two points must be fixed to make sure that the submitted manuscript specifically addresses the puzzling feature of multipartite viruses, that is the individual encapsidation of each of their genome segments.

We agree with Reviewer 3 that the separate encapsidation of genome segments is the most puzzling aspect of multipartite viruses. That is why our original manuscript focussed only on this aspect and did not consider the possibility of multiple encapsidation. As mentioned in our comments above, we have now extended the model to also consider joint encapsidation (Fig. 6).

Specific comments

-Line 39: "…segments must independently reach the same host…" the reference Di Mattia et al PNAS 2022 also appears appropriate when mentioning host-to-host transmission of segments. More importantly, though it may be implicit in the text, it is important that all readers understand that multipartitism means that each viral genome segment is encapsidated individually/separately. The following sentence may be slightly modified for "This type of genome segmentation, with each segment packaged separately, is termed multipartitism and entails substantial costs; most infections…"

We now define this aspect of multipartitism explicitly in lines 36-38. We now cite Di Mattia 2022 and we thank Reviewer 3 for pointing out this omission.

-Lines 55-57: I do not think that Gallet et al 2022 state that this benefit allowed the evolution of multipartitism. In multipartite viruses, what they report could be a benefit, this does not mean that it is what drove their evolution.

We have removed the citation from that sentence, and we now reference Gallet et al 2022 in the Discussion, in the context of experimental evidence for benefits to gene dosage adjustment in multipartite viruses (lines 614-620).

-Line 57-61: I do not understand how the example published by Ojosnegros and colleagues illustrates minimal conflict between different genome segments?

Ojosnegros et al test whether the fragments have a replication advantage over the ancestor (which could imply conflict) and find no evidence for this. The model in Iranzo & Manrubia subsequently assumes minimal conflict.

We have now changed this section of the text, to cite Iranzo & Manrubia for assuming minimal conflict, and to cite Ojosnegros for demonstrating a survival advantage to smaller capsids (lines 58-61).

-Lines 66-67: is it 100 viral genomes or 100 viral particles? There will be other similar questions below and it is very important that the authors are clear in the terminology they use when speaking about a viral genome, a genome segment, a virus, a virus particle. This is mandatory to clearly understand the issues of co-infection or MOI in this paper.

In the model referenced, each virion contains one genome, so the two are equivalent in that case. However, we feel it is clearer to say virion here, so we have changed the text.

We agree it is critical to carefully distinguish between viral genomes, genome segments, and virions.

Therefore, we have revised the main text to check each use of the word 'viral genome' or 'virion' to make sure that we are using the correct language each time.

-Line 109: "We examined the theoretical feasibility of the cheat hypothesis for the evolution of multipartitism and then tested it empirically": I do not see the empirical testing in this paper. Please explain.

We have changed the language to be more specific here (line 123). The text now reads '…tested both the assumptions and predictions of our model'. Figure 3 tests the assumptions of the model, i.e. whether the model can lead to multipartitism under plausible empirically derived parameter values. Figure 5 tests the predictions of the model, i.e. whether the cheating -> multipartitism hypothesis is consistent with known empirical trends. We have been clear to point out that neither of these are comprehensive tests (e.g. lines 474-482; 879-928), but that they are the best we can do with currently available data.

-Line 133-135: what is called a viral genome here? The cooperator monopartite, the sum of D1+D2 or only D1 or D2.

The cooperator encodes the complete genome. D1 and D2 are genome segments, that each encode a reduced version of the cooperator genome. We have changed the text to clarify here, and we have also added a reference to Fig. 1, which illustrates this visually (line 156).

We have also revised the rest of the manuscript to make sure we are consistent in this usage throughout.

-Line 154 and payoff matrix: in case of infection by one of each cheat or by the cooperator and one cheat, same payout "e" and "b" for each cheat, but this is likely a rare situation. Would it change something to the model output if payout would be different for D1 and D2?

We agree that this is a simplifying assumption made within the analytical model – instead, we address this question in the simulation. Our approach was to keep the analytical model simple enough that we could get analytical solutions, and to use the simulation to relax this assumption and others.

In the simulation, when the viral genome contains more than two genes (n_genes > 2), each of the partial cheats can have different payoffs, because each cheat can lack different numbers of genes. We find that when this is allowed, the population becomes multipartite more easily (Fig. S5). Furthermore, when the population splits into more 'uneven' multipartite segments (e.g. one segment encoding 7 genes and one encoding 1 genes, as opposed to both segments encoding 4 genes each), this makes the population more resistant to exploitation by full cheats that encode no genes (Fig. 5). Altogether, these simulation results suggest that uneven cheat payoffs actually make multipartitism easier to evolve via cheating, and hence our analytical results are potentially conservative in this regard.

-Figure 3: It appears bizarre to parametrize the model with data from polio, VSV, Rabies, and bunyawera, which are monopartite animal viruses. Of note however, for rhabdoviruses and bunyavirales, some multipartite species do exist in plants.

We agree that the ideal dataset here would be a virus that has both ancestral monopartite and derived multipartite forms, with accompanying experimental characterisation. However, we aren't aware of such a virus. Therefore, our goal in this figure is simply to illustrate the magnitude of within-cell advantage that viral cheats tend to get in reality, and to see whether these advantages are similar in magnitude to the advantages that our model requires in order for multipartitism to evolve.

To make this point clearer, we have now changed this figure to include both viral cheats whose values for key parameters are close to those required for multipartitism to evolve (this appears to be the case for all defective interfering genomes we've found), and viral cheats whose values are not (two phage cheats, which depend on short point mutations and are not defective interfering genomes) (Fig. 3).

-Line 233: "we allowed >2 viruses to co-infect cells": again, what means "viruses" in this sentence? (IDEM line 258)

We have changed to clarify viral particles.

-Lines 263-269: yes! but this largely depends on the benefit of cheats themselves (b and e) relative to that cooperator. What are the values leading to multipartitism, are they reasonable?

We tested this in Figure 3 using the parameters from the analytical model. These are more directly comparable to experimental data, which is inferred from pairwise experiments. Our simulation implements the analytical parameters in a way that allows for more than two genes.

We have changed the text here to clarify (lines 347-353). We have also added in additional in-text references to Fig. 3, in addition to Fig. S3, which presents a parameter sweep to ensure that our simulation is consistent with the analytical model, even when adding extra biological details.

-Same lines: I am wondering how or when "e" can be considered a group benefit? when e>c, a or d. But could it be when e>b? Perhaps this is indicated somewhere.

In the simulation, 'e' is a parameter that multiplies the productivity of a host cell according to how many complete sets of genomes come from a multipartite virus, instead of a monopartite virus. Hence in the simulation, group benefits to multipartitism can occur when e > 1. We have now added this explicitly into the description of the simulation lifecycle (lines 952-953).

-Figure 4: why does crash occur only at high MOI? This could be explained either here or in the discussion.

We have now generated a more detailed dataset for this figure, at a higher resolution (Fig. 4). We now find that these populations do persist, just at a very low abundance. All code required to reproduce these results is available at https://osf.io/pbe4n/?view_only=9a4a2802f2564131b94a323fa21bbcbe and is fully commented. This includes parameter values used (including random seed values for reproducibility) as well as analysis scripts in R.

Lines 299-301: I have a problem with the data on which this is based. What exactly is important? Is it the rate of production of defectives or their capacity to accumulate in the system? These may be totally distinct, and the data collected here could be biased in that sense. Let's suppose that all viruses rampantly produce tones of defectives but only in some cases one defective "species" becomes sufficiently frequent to be detected. The collectively huge amounts of myriads of distinct defectives would be mostly overlooked (unless long read sequencing is analyzed). To date, only scattered studies are available, and most do look at defectives that accumulate, not at their rate of production. But perhaps the two are linked. I don't know. I acknowledge the fact that the authors are already cautious about this conclusion of Figure 5, but more discussion is required. When going more locally in the viral clades, some closely related viral taxa have both mono and multipartite member species and so the correlation shown in Figure 5 appears to depend on the chosen scale.

We agree with Reviewer 3 that it is critical to distinguish between defective viral genomes and defective interfering viral genomes, since only the latter count as evolutionary cheats. We have made this point ourselves in previous work (Leeks et al 2021 https://www.nature.com/articles/s41467-021-27293-6). To conduct this analysis, we have only included studies in which it was demonstrated that a viral genome is both defective and interfering (i.e. accumulates at the expense of the wild-type viral genome). This is consistent with the definition of defective interfering genome used in the literature, that is considered a type of viral cheat (Huang and Baltimore 1970; Vignuzzi & Lopez 2019; Leeks et al 2021).

To clarify this, we have now changed the text in Appendix 4 where we describe how we decided which studies to include for this dataset: "To ensure that we were only including viral cheats, we kept only records identified as defective interfering genomes, defined as truncated viral genomes generated from the wild-type, that were not able to infect cells on their own, and that interfered with the accumulation of the wild-type virus (Huang & Baltimore, 1970; Leeks *et al.*, 2021). Overall, this resulted in a database of 49 viral genera known to produce defective interfering genomes." (lines 996-998).

We agree that the taxonomic scale matters for Figure 5 (now Fig. 7). We did not choose Realm arbitrarily. We chose to use viral Realm because this is the only grouping that is generally accepted as reflecting an independent evolutionary origin. Hence we felt that any more fine-grained division would risk treating phylogenetically related data points as independent data points (Felsenstein 1985).

-Line 331: what means "viral genome" here?

Changed

-Lines 332-336: It seems that multipartite viruses with 2 or 3 segments can evolve at MOI that are even compatible with values reported in animal viruses, or phages, and yet they do not exist there. Please comment on this in the discussion, specifically for multipartite viruses with a low number of segments.

We have added a new section in the Discussion on why we think multipartite viruses are more common in plants (lines 639-686). We think that an important factor is the variation in MOI, which could be lower in plant viruses. This would be consistent with the fact that viral cheats in the form of satellite viruses often transmit between hosts in plant viruses, but not in animal viruses or phages (Leeks et al 2021). We also think it's important to be relatively conservative in making predictions here, since there are some examples of multipartite viruses in animals (e.g. http://dx.doi.org/10.1016/j.chom.2016.07.011), and there may be more to be discovered.

-Line 354: I do not understand what means "identify conditions under which multiple different types of cheat are possible"

Our model predicts that multipartitism evolves more easily when there are a larger number of cheatable genes in the genome (Fig. S5).

We have now changed this sentence to clarify our meaning (lines 563-564).

-Lines 363-364: this is rather a matter of number of generations, the time is not key,
The authors seem to consider this could arise in a few generations but then the evolved multipartite would meet the monopartite ancestor all the time in the outworld.

We agree, and have changed this section to clarify that the number of generations is the key factor (line 642).

The question of multipartite viruses meeting monopartite ancestors is an interesting one, but it is beyond the scope of the current manuscript, and our models do not directly test this. Group benefits to multipartitism could play a role in determining the outcome of these meetings; we discussed this possibility in lines 624-626, in our new section on group benefits.

-Lines 365-366: Could the authors be more specific on the estimate of transmission bottleneck comparison between plants and animals because they may not be so different, there are not a lot in the literature and so actual numbers could be discussed.

We have revised this section and now include some more estimates from the literature (lines 649-686). We agree it is hard to be conclusive given the paucity of data, and we have tried to write our Discussion to reflect this.

-Lines 384-385: Do the authors imply that reversion to monopartite is possible? or that monopartite related virus species would outcompete those that have evolved multipartitism. This point should be explicit.

Here we mean that monopartite may outcompete multipartite species over longer timescales. We changed the text to clarify (line 591).

We know of only two examples that could be analogous to multipartite reverting to monopartite (Shirogane et al 2012 http://www.nature.com/ncomms/journal/v3/n12/full/ncomms2252.html and Sachs and Bull 2005 http://www.pnas.org/content/102/2/390).

-Lines 415-421: Yes, segmented viruses are concerned and encapsidating together several segments would facilitate co-infection. Both specific and non-specific encapsidation of segments exist in segmented viruses, and this may have a distinct impact on "co-infection". It would be interesting to see how a multipartite virus would do when competing with similarly segmented genomes but packaged together. It is even a key question for the conclusion of this manuscript.

We have now included an extension to the model in which we model segmented viruses (Fig. 6) – see earlier reply.

-Lines 415-418: What would lead evolution into multipartite forms rather than segmented forms? Please comment.

We now discuss this question explicitly in our new section of the Results focusing on segmentation (Fig. 6; lines 441-466), and in our new section of the Discussion (lines 631-758).

-Lines 423-430: This goes more and more towards segmentation (unfortunately) rather than multiple encapsidation and separate propagation of segments or genome fragments. Please see the related Major comments.

Please see our response to earlier comments.

-Lines 439-440: A more optimistic view would present those conflicts has creating opportunities for the transient appearance of more complex genetic systems that may then unveil emerging beneficial properties. Such beneficial properties (which do not exist for monopartite ancestors) could in some cases allow the maintenance of multipartitism. Multipartitism would thus evolve from conflicts but be maintained by emerging properties in the system.

We agree with this viewpoint. We now explicitly discuss this in our new section on benefits to multipartitism (lines 594-629). We think that these kinds of emergent benefits could play an important role in the maintenance of multipartitism in particular, and we emphasise this in the main text (e.g. lines 595-597). We think that it is valuable here to distinguish between different types of mechanistic advantage to multipartitism (e.g. those that are determined by physical properties of virions, vs those that arise as new adaptations facilitated by the emergence of multipartitism), and we have structured this section of the discussion along these lines.

-I do not understand Figure S7 (that is mistakenly labelled Figure S6 I think). Either the legend should be re-written, or the Figure organized differently. What are the "0" and "1" down the graph? What do the 1 to 10 numbers on the top indicate?

We have removed this figure and replaced it with a new main figure (Fig. 5), to accompany the new sections on benefits to multipartitism.
