# Supplementary material for: Cheating leads to the evolution of multipartite viruses
Source: PLoS Biol. 2023 Apr 24;21(4):e3002092. doi: 10.1371/journal.pbio.3002092 (PMC10159356; doi:10.1371/journal.pbio.3002092)
Supplement: Attachment — Submitted filename: multipartite_reviewer_reply_2.pdf [file pbio.3002092.s003.pdf]

REVIEWERS' COMMENTS:

Reviewer #1:
[identifies himself as Eugene V Koonin]

I found the revisions made by the authors to be satisfactory and a substantial improvement, making the paper highly convincing.

In particular, by including an interesting discussion of potential group benefits of multipartitism, I believe the authors address the comments of reviewer 2 regarding biases in the original presentation.

I have no further suggestions.

We thank Dr Koonin for the kind comments and careful reading of the manuscript.

Reviewer #2:
[identifies himself as Bram van Dijk]

Dear authors,

Your revisions are really thorough, to the point that I really enjoyed reading your responses to even my most hard criticism. I also am very happy with the new figures that were added to get a little bit deeper into the topic.

I have no further comments, and I am in favour of publication.

Best,

Bram

We thank Dr van Dijk for the kind comments and careful reading of the manuscript.

Reviewer #3:

Leeks et al 2022 R2

I do consider that the authors have mostly satisfactorily responded to my comments. Therefore I have no additional comments, but some very minor points

-Line 37 (but also elsewhere including the Abstract): I am not sure what "independently" or "separately"' means here and in the abstract. For nanoviruses, that segments can move separately within plant and also between plants have some experimental support. In rift valley fever bunyavirus, that distinct particles containing incomplete sets of segments can- also move separately cell-to-cell and even into insect vector, co-infect and reconstitute a full infection, also received experimental support recently. However, it does not imply that they HAVE TO move separately or independently to successfully infect. This sentence can be slightly modified just to nuance this point.

We agree that the previous phrasing didn't fully reflect the nuance of the situation. We have now changed line 37 (now 38) in the Introduction and line 8 in the Abstract, from "separately reach the same host" to "are present within the same host".

-Line 45-50: I am still troubled by the fact that the authors want to generalize their findings or investigation to plasmids, integrated phages and endosymbionts. Their model applies to viruses and I am not sure I can clearly see how and whether it could similarly apply to these other organisms, whether they are really "analogous" type of genome fragmentation. I understand that the authors really wish to discuss this point and so I suggest to remove it from the Introduction and rather keep the part in the discussion. Also, the authors may want to delete the terms -"analogous" types of genome fragmentation- or -"similarly" fragmented-. "Other types of genome fragmentation" and "are also fragmented" may be more appropriate

We agree that our models are primarily inspired by multipartite viral biology. Hence, the extent to which these other examples are captured by our model will depend on the details of the biology in each case. For this reason, in lines 513-531 of the Discussion, we explicitly consider the extent to which each of these cases might be analogous to multipartite viruses as treated in our models. For example, we suggest that fragmented temperate phages may be more similar to multipartite viruses when transmitting horizontally (via lysis), but may be less analogous when transmitting vertically (via bacterial cell division). Because our model is centered on social conflict, we think that the fundamental mechanism could well apply outside viruses; indeed, examples of cheating are well documented in endosymbionts, temperate phages, and plasmids. Therefore, our goal here is to highlight that cheating could be useful for explaining genome fragmentation in these potentially analogous systems, even if we do not explicitly model each system in this paper.

To better clarify this point, we have:

- Replaced 'analogous types of genome fragmentation' and replaced it with 'other types of genome fragmentation' (line 47)
- Replaced 'similarly fragmented' with 'are also fragmented' (lines 514-515).

-Line 52: focus or rely ?

Rely – we thank Reviewer 3 for catching this typo.

-Line 326 Section on segmented viruses: It remains unclear to me if multipartitism can evolve and get fixed when segmented viruses (encapsidating segments together) are allowed. Could multipartite outcompete segmented in some instances ?

This is a good point. Our model finds conditions under which segmented evolves easily but multipartite does not (Fig. 6). This would suggest that there are cases when segmented outcompete multipartite. However, we find that when both segmented and multipartite are possible, both types coexist within the population. Hence, we don't explicitly find cases in which multipartite viruses outcompete segmented viruses. We agree that this is an interesting question, especially given the surprising prevalence of multipartite compared to segmented viruses.

In our view, we think that the best way to directly address this question would be to restructure the model to allow genetic linkage between the strategy for encoding shared viral genes (i.e. cheat or cooperator) and the transmission strategy (i.e. encapsidate separately or together). Clearly, this would involve many additional assumptions about how encapsidation occurs, how cheating arises, and the extent to which these outcomes are under virus or host control.

We think that this would be a valuable extension to our modelling, but that it is beyond the scope of the current manuscript. To spur future work in this direction, in the Discussion we consider the limitations of our approach to modelling segmented viruses, including explicitly pointing out this possible extension (lines 501-511).

-I have the feeling that many predictions from Table 1 are not supported by available data but, I agree with the authors that most have not been formally tested and these predictions have the merit to now exist and, in that sense, I agree they are interesting.

We agree that these questions are very open. We are hopeful that recent experimental and genomic advances in the study of multipartite viruses will make them amenable to testing in the near future.

-Lines 487-488: Transmission by insect is most often (if not always) shown to induce strong bottlenecks. The sentence in these line apparently states the contrary? "….resulting in relatively wider bottleneck" relative to what ? The examples given appears to be chosen to make the authors point, not reflecting the state of the art. There are vector transmitted animal viruses with large bottlenecks at transmission (mosquito transmitted for example). The bottlenecks during between host transmission of IAV and other HIV / HCV are not always small. In addition, the value given for Cauliflower mosaic virus, is not a value for between host transmission but the value for intra-plant cellular MOI ? Values for vector transmission bottlenecks for potyviruses and cucumoviruses are smaller I think.

Perhaps, one way out of this could just be to argue that these are values from single aphid vector and that the bottlenecks may be enlarge by several aphids visiting the plants in the field (this type of information is unfortunately poorly documented but the possibility of several aphids visiting the same plant has been often mentioned)

We thank Reviewer 3 for their clarifying comments on vector-borne bottlenecks here. In our view, there is insufficient evidence for systematic comparisons between these modes of transmission. Hence, our goal was to suggest some speculative hypotheses for the apparent over-representation of multipartite viruses among plants, without definitively asserting that any of these are the primary reason. Even if the reasons why are not entirely clear at this stage, it does seem that cheats are able to transmit from host to host more easily in plant viruses, as reflected by the high prevalence of satellite viruses in plants. Hence, the apparent abundance of multipartite viruses in plants could be consistent with cheating driving multipartitism.

We have now streamlined and softened the language in this section of the Discussion, to better reflect the state of the art, and the speculative nature of this section (lines 490-504). We have additionally incorporated Reviewer 3's suggestion regarding transmission via several vectors to the same plant (lines 498-499), and to support this we have cited a recent experimental paper showing that such sequential transmission can allow reconstitution of multipartite viral genomes (Di Mattia et al 2022). We thank Reviewer 3 for their useful comments, that have improved this section of the Discussion.
